# Effect of traditional Chinese medicine on outcomes in patients with mild/moderate chronic obstructive pulmonary disease: study protocol for a randomized placebo-controlled trial

**DOI:** 10.1186/1745-6215-13-109

**Published:** 2012-07-16

**Authors:** Wang Minghang, Li Jiansheng, Li Suyun, Wang Haifeng, Yu Xueqing, Zhang Hailong

**Affiliations:** 1 Department of Respiratory Diseases, The First Affiliated Hospital of Henan University of Traditional Chinese Medicine, Zhengzhou, Henan Province, 450000, People’s Republic of China; 2 Institute of Geriatrics, Henan University of Traditional Chinese Medicine, Zhengzhou, Henan Province, 450008, People’s Republic of China

**Keywords:** Chronic obstructive pulmonary disease, Traditional Chinese medicine, Treatment, Clinical trials

## Abstract

**Background:**

Chronic obstructive pulmonary disease (COPD) has become a major public health problem worldwide because of its high and increasing prevalence, morbidity, and mortality. Little attention has been paid to earlier stages of COPD or before it has developed. Reportedly, TCM may have some advantages in relieving symptoms and reducing the incidence of COPD exacerbations. We postulate that patients with COPD will benefit from therapy with TCM treatment according to syndrome differentiation.

**Methods and design:**

A prospective, multi-center, double-blinded and randomized controlled method will be used to test the therapeutic effects of TCM treatment according to syndrome differentiation. A total of 504 patients will be enrolled into this study with 252 in each treatment group. Patients will receive medication according to their assigned group. TCM for COPD will be administered twice daily over 52 weeks, and all patients will follow the treatment program for 52 weeks. The FEV_1_ and exacerbations will be used as the primary outcome measures. The quality of life and the Modified Medical Research Council (MMRC) Dyspnea Scale, and the 6-min walk test (6MWD) will be used as the secondary outcome measures.

**Discussion:**

We postulate that patients with COPD will benefit from therapy with TCM treatment according to syndrome differentiation.

**Trial registration:**

This study is registered at ClinicalTrials.gov, NCT01486186

## Background

Chronic obstructive pulmonary disease (COPD) has become a major public health problem worldwide because of its high and increasing prevalence, morbidity and mortality [1,2]. A recent report estimated that the global prevalence of COPD (stage II or over) is about 10% (12% in men and 9% in women) [3]. In China, COPD affects 8% of the population aged 40 and above [4], and is one of the top diseases in the World Health Organization’s ranking of burden of diseases [5]. It is therefore necessary to develop a reliable intervention strategy for the prevention and management of COPD to reduce the burden of this disease.

Current interventions for COPD (such as smoking cessation, rehabilitation, nutrition support, drug treatments and psychosocial aids) are mainly on a case-by-case basis among patients who have already developed moderately or severely symptomatic COPD, while little attention has been paid to earlier stages of the disease or before it has developed. Although there has been some progress with case-based or hospital-based interventions [such as improvements in exercise capacity, forced expiratory volume in 1 s (FEV_1_) and health-related quality of life, fewer hospital admissions and acute exacerbations, and lower all-cause mortality] [6-9], such efforts for the prevention of COPD are limited among the general population in China. Few patients are diagnosed until they develop severe symptoms and signs, and pulmonary function tests are not routinely performed.

Traditional Chinese medicine (TCM) is very popularly used for COPD in Asia, and reportedly TCM may have some advantages in relieving symptoms, reducing the incidence of COPD exacerbations and improving the quality of life for COPD patients [10,11].

We report in this article on a multi-center, randomized, double-blinded, placebo-controlled trial of traditional Chinese medicine treatment with syndrome differentiation to test the hypothesis that patients with COPD will benefit from the therapy with TCM.

### Objective

To determine whether treatment with TCM with syndrome differentiation reduces exacerbation frequency.

## Methods and design

### Study design

This clinical trial has a multi-centered, randomized, double-blinded, placebo-controlled design. The study design will integrate rigorous, contemporary clinical research methodology in accord with the principles set out in the Declaration of Helsinki and the Good Clinical Practice guidelines according to the theory that guides the appropriate use of TCM in clinical practice. Reporting will be guided by the CONSORT statement [12,13]. Subjects will be enrolled at 12 hospitals: (1) the First Affiliated Hospital of Henan University of TCM, (2) TCM Hospital of Xinjiang Uygur Autonomous Region; (3) the First Affiliated Hospital of Anhui University of TCM; (4) Xiyuan Hospital of the China Academy of Chinese Medical Sciences; (5) Jiangsu Province Hospital of TCM; (6) Shanghai Shuguang Hospital affiliated with Shanghai University of Traditional Chinese Medicine; (7) the Second Affiliated Hospital of Guiyang College of TCM; (8) Jilin Academy of Traditional Chinese Medicine; (9) Shanxi Sheng Zhongyi Hospital; (10) the First Teaching Hospital of Tianjing University of TCM; (11) the First Affiliated Hospital of Guangzhou University of TCM; (12) Jiangxi Province Hospital of TCM. This study will be conducted at 12 sites in China. Twelve hospitals in China will randomly assign 504 patients with COPD at a ratio of 1:1 into the TCM treatment or matching placebo group. Consenting eligible participants will be enrolled for 2 weeks and will be required to attend seven visits in total. The trial is designed in two phases, 52 weeks of treatment with either TCM for COPD or placebo granules, and 52 weeks of follow-up.

### Study duration

Ongoing recruitment will occur for a maximum period of 18 months (between July 2011 and December 2013) or until a sample of 504 individuals has been randomized.

### Subjects

A total of 504 participants will be recruited through local advertising and doctor referrals from hospital outpatients and general practice clinics. Interested participants can telephone or email the trial coordinators at the corresponding sites for further information. Participant information and consent forms will be sent to interested individuals to read over prior to scheduling their first visit.

### Inclusion criteria for participation in the trial

The inclusion criteria are as follows: (1) a confirmed diagnosis of mild/moderate COPD; (2) males and females aged between 40 and 80 years; (3) meeting the TCM diagnostic criteria (syndrome differentiation includes lung *qi* deficiency, pulmonosplenic *qi* deficiency and insufficient lung and kidney *qi* syndromes); (4) not having participated in other interventional trials in the previous 1 month; (5) giving written informed consent to participate.

### Exclusion criteria

Exclusion criteria include: (1) pregnant or breast-feeding women; (2) any psychiatric condition rendering the patient unable to understand the nature, scope and possible consequences of the study; (3) complication of severe heart failure (NYHA heart function class IV) or unstable hemodynamics; (4) complication of bronchial asthma, bronchiectasis or active tuberculosis, obliterative bronchiolitis or diffuse pantothenic bronchiolitis, pneumothorax, pleural effusion or pulmonary embolism; (5) complication of neuromuscular disorders affecting respiration or of tumors; (6) complication of serious hepatic and renal diseases; (7) long periods of bed rest; (8) use of oral or parenteral corticosteroids in the 1 month before the first visit; (9) having immunodeficiency; (10) participating in other trials or being allergic to the used medicine.

This study is to be conducted in accordance with patient protection as outlined in the Declaration of Helsinki and approved by the appropriate Institutional Review Boards. Each participant will sign the written informed consent form before undergoing any examinations or study procedures, in compliance with Good Clinical Practice. Patients who initially meet the eligibility criteria are then to complete the additional baseline testing and will be randomized into either the treatment or control group.

### Ethics issues

This study has been approved by the Ethics Committee of the First Affiliated Hospital of Henan University of TCM (no: 2011HL-034). Each participating center obtained local institutional review board approval. All study participants will sign the written informed consent prior to participation.

### Interventions

Eligible patients will be randomized to one of the two arms: receiving a placebo or a Chinese medical herb. An herbal extract will be given twice daily for 52 weeks. There are three recipes for the three traditional Chinese syndromes: lung *qi* deficiency, pulmonosplenic *qi* deficiency, and lung and kidney *qi* insufficiency syndromes. The experimental group will receive three types of TCM: Baofei granules, Bufeijianpi granules and Bufeiyishen granules. The placebo comparison group will receive placebo Chinese medicine in addition to best care according to the clinical guidelines for COPD patients.

Randomization of subjects will occur centrally using a random number generator and will be stratified by syndrome differentiation of TCM (a syndrome, as related to illnesses in Western medicine, is composed of a set of signs and/or symptoms classified by Traditional Chinese Medicine practitioners. TCM syndrome differentiation is based on symptoms and helps to identify a disease subset).

### Randomization and allocation

Treatment allocation occurs when the study participant meets the inclusion criteria and signs the informed consent form. The teletherapist will then register the participant into the database, which in turn asks if they are ready to be randomized. After the teletherapist enters “yes,” the site-specific randomization program behind the form displays the participant's group assignment number (placebo versus Chinese medical herb). Site-specific randomization lists will be computer-generated (i.e., generated by an individualized basic visual code program) and concealed from the researchers by a senior data manager who is not involved in the study. This information will remain confidential and will not be shared with the study sites, in concordance with the CONSORT guidelines. This trial uses a prospective, randomized, double-blinded design. The allocation list will be protected by password access files and held by an independent non-investigator. In the event of an emergency medical situation, the individual’s randomization code and group allocation can be identified.

### Sample size

The sample size calculation is based on the annual decline of mean forced expiratory volume in 1 s (FEV_1_) in COPD subjects. The previous study showed that the mean rate of change in FEV_1_ was a decline of 51 ml per year in patients with TCM treatment and 131 ml per year in patients with usual care with mild/moderate COPD. According to the sample size of the estimation formula $n=2{\left[\frac{(u_{a}+u_{\varphi })\sigma }{\delta }\right]}^{2}$, n1 = n2 = 2(1.64 + 1.28)^2^ × 0.28^2^/0.08^2^ = 209. It is estimated that a sample size of 209 subjects per group will be required, considering 20% drop out and exit. This results in 504 in total. Intention-to-treat analysis will be applied to minimize bias due to drop-outs.

### Outcome measurements

#### Primary outcome measure

##### Pulmonary function (FEV_1_)

Pulmonary function (FEV_1_) will be evaluated prior to treatment, at weeks 26 and 52 of the treatment phase, and weeks 26 and 52 of the follow-up phase.

##### Exacerbation

Frequency of exacerbations of COPD will be evaluated at weeks 26 and 52 of the treatment phase, and weeks 26 and 52 of the follow-up phase. If the interval between two onsets of acute exacerbation is within 5 days, it can be counted as one period of acute exacerbation.

#### Secondary outcome measure

##### Dyspnea

The Dyspnea Scale Questionnaire, which was first developed by the British Medical Research Council (MRC) and later revised by the American Thoracic Society (MMRC), will be observed and recorded prior to treatment at weeks 13, 26, 39 and 52 of the treatment phase, and weeks 26 and 52 of the follow-up phase.

##### Quality of life (QOL)

The secondary outcome measures will be QOL improvement assessed by using the COPD Assessment Test (CAT) [14] and short-form 36-item questionnaire (SF-36) [15]. They will be observed and recorded prior to treatment, at weeks 13, 26, 39 and 52 of the treatment phase, and weeks 26 and 52 of the follow-up phase.

##### The 6-Min walking distance test (6MWD)

In patients with COPD, the 6 MWD is a good predictor of health-care utilization. The 6MWD will be observed and recorded to prior treatment, at weeks 13, 26, 39 and 52 of the treatment phase, and weeks 26 and 52 of the follow-up phase.

#### Adverse event reporting

pirometry at each site will be conducted using SpiroUSB™ (CareFusion) and Spida5® software. Routine calibration and standard operating procedures will be uniformly undertaken at each site. Participants will be advised not to use respiratory medications for several hours prior to their spirometry testing. Two sets of measurements will be taken at each visit, pre-bronchodilator and post-bronchodilator (post 400 μg of inhaled salbutamol).

Any adverse events will be listed in the trial record and followed up to completion by the trial coordinators and respiratory physicians. Adverse event details will be scored using a six-point scale: 0 = none, 1 = minimal, 2 = mild, 3 = moderate, 4 = severe and 5 = extremely severe. Participants will be able to report adverse events anytime throughout the trial and will receive advice accordingly. Serious adverse events will be reported to the reviewing Human Research Ethics Committee (HREC) and site HRECs within the timeframe specified by the lead HREC.

Throughout the study period, trial coordinators will contact the participant’s usual treating doctor to record relevant data on presentations and exacerbations.

To assist with outcome documentation and medication compliance, participants will be given a participant diary for the duration of the trial. They will be asked to record trial medication and relief medication usage as well as any new medications they commence during the trial.

Participants may withdraw from the study for any reason at any time without repercussions. They will only be withdrawn by investigators if it is deemed medically unsafe for them to continue. Dropouts will not be replaced.

### Statistical analysis

Descriptive statistical analysis will be performed for all the study variables. We will calculate the mean, median and standard deviations for quantitative variables, and the absolute and relative frequency for qualitative variables.

We use SPSS 19.0 (license no.: 6f1d84c801f1e6010dc, SPSS Inc., Chicago, IL, USA) to statistically analyze the data. An intention-to-treat analysis will be applied using the Last Observation Carried Forward (LOCF) method. Analysis of covariance with baseline as covariate will be used to assess differences in treatment outcomes between the two groups at each of these time points. To correct for inflated risk of type 1 error, multiple comparison procedures suggested by Ludbrook et al. will be used [16]. The use of relief medication during the trial will be investigated for its effect on the outcome measures by using it as a covariate in the statistical analysis. A Data Safety Monitoring Board has been established to assess the progress of the trial, particularly safety endpoints.

## Discussion

Chronic obstructive pulmonary disease (COPD) remains a major public health problem. The aim of COPD treatment should be to decrease symptoms and exacerbations, reduce the risk of hospital admission, increase lung function and improve QOL. Recently, the approach to therapy has focused on symptomatic relief.

Traditional Chinese medicine (TCM), as a system of medicine, has been a form of health care in China and its neighboring countries for several thousand years. Some TCM therapy, such as Tanreqing injections, has been reported to be of some benefit to COPD patients, and could improve the Chinese medical signs and symptoms in patients with AECOPD, reducing airway inflammation and airway mucus hypersecretion [17,18]. Others improve lung function and relieve airway inflammation in patients with stable COPD [19].

Currently, there are no TCM treatments involving syndrome differentiation therapy for COPD patients; therefore, we designed this study. The study will be a large-sample, multicenter, long-term chronic obstructive pulmonary disease study. The data gathered will shed new light on traditional Chinese medicine, especially for COPD treatment.

## Trial status

At the time of manuscript submission, we had recruited 150 patients but not completed the patient recruitment.

## Abbreviations

COPD, Chronic obstructive pulmonary disease; CAT, COPD assessment test; FEV1, Forced expiratory volume in 1 s; FVC, Forced vital capacity; GOLD, Global initiative for chronic obstructive lung disease; LOCF, Last observation carried forward; QOL, Quality of life; RCT, Randomized controlled trial.

## Competing interests

The authors declare that they have no competing interests.

## Authors’ contributions

W-M participated in the design of the study and the intervention. LJ-s was involved in the design of the study and participated in reviewing the manuscript. LS-y was involved in the design of the intervention and participated in reviewing the manuscript. W-H was involved in drafting the manuscript and writing it. He participated in the design of the study and the intervention. YX-q was involved in the design of the study and participated in reviewing the manuscript. ZH-l was involved in the design of the study and participated in reviewing the manuscript. All authors read and approved the final manuscript.
